# The Etiologic Diagnosis of Chronic Cough in Patients Attending a University Affiliated Cough Clinic

**Published:** 2011-06-01

**Authors:** Sh Firoozbakhsh, S Seifirad, E Safavi, H Vahedi, M Zahedpouranaraki

**Affiliations:** 1 Pulmonary and Critical Care Research Center,Tehran University of Medical Sciences, Tehran, Iran

**Keywords:** Chronic cough, Epidemiology, Diagnosis

Dear Editor,

Cough is one of the most common complaints for which patients seek medical attention.[1] In order to determine the etiologic diagnosis of chronic cough in our patients and to find out our success rate in the diagnosis and treatment of these patients, 100 consecutive patients with the chief complaint of chronic cough, who attended the cough clinic in our center,were evaluated; each patient was followed for at least 6 months. The diagnostic procedure was based on the anatomic protocol for diagnosing chronic cough designed by Irwin.[1][2][3] A combination of history, physical examination, and laboratory findings to evaluate possible causes of cough were considered. The suspected cause could be confirmed if the cough resolved or significantly improved after a trial of diagnosis specific treatment.

Fifty-five percent of patients were female and 45% were male. Their mean± SD age was 45.13±17.43.Seventy-one percent were non-smokers, 13% passivesmokers,9% ex-smokers, and only 7% of them were active smokers. Two patients were on Angiotensin Converting Enzyme Inhibitor (ACE-inhibitor) drugs.Sixty-three percent of patients complained of postnasal discharge, while 56% of them had dyspnea, and 6 patients suffered from heart burn.

The etiology of chronic cough was determined in all of our patients. In 15% of patients, two causes were responsible simultaneously. The final diagnoses are shown in Table 1. The most common etiologies of chronic cough were upper airway cough syndrome (due to Post Nasal Discharge (PND)), asthma and Gastro-Esophageal Reflux Disease (GERD).[1][3][4][5][6][7][8] However, a number of other etiologies should be considered in patients presenting chronic cough. Postinfectious cough, complication of drug therapy (i.e.ACE inhibitors), airway disease (chronic bronchitis,bronchiectasis, neoplasm, foreign body), pulmonary parenchymal disease (interstitial lung disease, lung abscess),are samples of chronic cough etiologies with lower prevalence.

As it is evident from this study, by following the anatomic diagnostic protocol, a cause for cough can be found in almost all cases. Thus, systematic evaluation remains the most effective approach to chronic cough and diagnosis-based treatment is the best way to manage it. Fifty-five percent of patients in our study were female. It has been shown that women are more likely than men to develop chronic cough, because they have more sensitive cough reflex than men.[9] In our study, PND syndromes and asthma either alone or both together accounted for the most cases of chronic cough, and this is in accordance with the results of other studies in this field.[3][4][5] Underlying etiologies of PND include allergic, perennial nonallergic and vasomotor rhinitis, acute nasopharyngitis,and sinusitis.[1]

Asthma is the second leading cause of persistent cough in adults and the most common cause in children.[10] It is commonly accompanied by episodic wheezing and dyspnea, but it can also be the sole manifestation of cough variant asthma. Chronic bronchitis was not a common cause for cough in these patients, as was predictable with respect to previous studies.[3][4][5]

GERD is often reported to be the second or third most common cause of chronic cough, (30-40% of patients with chronic cough).[1][4][5][7] But in our study,GERD was considered as the etiologic diagnosis in only 1% of our patients which is not compatible with the results of other studies.[3][4][5] It seems that the awareness of primary care physicians is responsible for the low prevalence of GERD as the etiology of cough in our referral cough clinic.

Bronchiectasis was one of the prevalent causes of cough in our patients. This relation can be due to common occurrence of this disease in our community or due to undergoing diagnosis of it in primary care centers, probably because High Resolution Computerized Tomography (HRCT) is not commonly used by our physicians in primary care centers. Tuberculosis was found in 3% of our patients. With respect to this study, we cannot recommend that every patient presenting chronic cough as the sole symptom should be rigorously investigated for tuberculosis.

ACE inhibitor-induced cough was diagnosed in only one patient despite the frequent use of these drugs. It seems to be due to awareness of clinicians who change the drug in suspected cases as soon as possible. In our study, PND syndromes and asthma,either alone or in combination with other etiologies were among the most common causes of chronic cough. It should be remembered that more than one factor can be responsible for chronic cough in a given patient. In evaluating chronic cough, it is prudent to apply diagnostic guidelines step by step and treat underlying cause(s) accordingly.

**Table 1:** Final Diagnosis of 100 patients with chronic cough

**Final diagnosis**	**Patients No.**
PND a Syndrome	60
Asthma	10
PND and Asthma	11
Bronchiectasis and PND	4
Bronchiectasis	3
T.B b	3
Chronic Bronchitis	3
GERD	1
ACE inhibitor	1
Miscellaneous c	4
Total	100

^a^ PND: Post Nasal Discharge, COPD: Chronic Obstructive Pulmonary Disease

^b^ TB: Tuberculosis, GERD: Gastro-Esophageal Reflux Disease

^c^ Lung cancer 1%, PND syndrome and chronic Bronchitis 1%, Sarcoidosis1%, Idiopathic Pulmonary Fibrosis (IPF) 1%, and PND syndrome with CHF 1%.
